# Supplementary material for: Thalamus and claustrum control parallel layer 1 circuits in retrosplenial cortex
Source: eLife. 2021 Jun 25;10:e62207. doi: 10.7554/eLife.62207 (PMC8233040; doi:10.7554/eLife.62207)
Supplement: Supplementary file 1. — contains Table 1, Table 2, and Table 3. Table 1 presents mean ± SEM values of intrinsic properties of LR, L3 RS, and L5 RS cells. Table 2 presents the exact p-values evaluating differences between the three cell types for each intrinsic property examined. Source data files for these intrinsic properties are found in Figure 1—source data 1. Table 3 presents exact parameter distributions used for the model (see ‘Modeling the Presynaptic HD Population’). [file elife-62207-supp1.docx]

|  | **LR** | ***n*** | **L3 RS** | ***n*** | **L5 RS** | ***n*** |
| --- | --- | --- | --- | --- | --- | --- |
| Postnatal age at time of recording (days) | **70.32** ± 3.65 | *132* | **78.00** ± 6.06 | *80* | **79.74** ± 7.37 | *42* |
| Input resistance (MΩ) | **478.10** ± 16.09 | *122* | **163.31** ± 10.81 | *78* | **139.07** ± 10.83 | *37* |
| Input capacitance (pF) | **32.10** ± 1.20 | *122* | **95.53** ± 3.17 | *78* | **107.65** ± 5.73 | *37* |
| Membrane time constant (ms) | **14.13** ± 0.36 | *122* | **13.78** ± 0.52 | *78* | **13.42** ± 0.75 | *37* |
| Action potential threshold (mV) | **-40.16** ± 0.33 | *120* | **-40.89** ± 0.33 | *78* | **-38.78** ± 0.75 | *38* |
| Action potential amplitude (mV) | **62.97** ± 0.82 | *120* | **76.77** ± 0.91 | *78* | **73.16** ± 1.92 | *38* |
| Action potential width (ms) | **0.70** ± 0.02 | *120* | **0.90** ± 0.04 | *78* | **1.04** ± 0.07 | *38* |
| Spike frequency adaptation ratio | **1.27** ± 0.04 | *120* | **2.55** ± 0.14 | *78* | **2.50** ± 0.21 | *38* |
| Latency to first spike (ms) | **497.90** ± 22.57 | *104* | **227.54** ± 24.99 | *70* | **179.11** ± 34.26 | *24* |
| Rheobase (pA) | **36.88** ± 4.02 | *104* | **85.58** ± 6.7 | *70* | **121.20** ± 16.01 | *24* |

**Supplementary Table 1. Intrinsic cell properties.**

**Supplementary Table 1. Intrinsic cell properties.**

Values are mean ± SEM. Ns are reported individually for each intrinsic property and cell type. All p-values for intrinsic property comparisons are reported immediately below in Supplementary File 1 – Supplementary Table 2. See Figure 1 – Source data 1 for source data.

**Supplementary Table 2. P-values for intrinsic property comparisons across cell types.**

|  | **LR v. L3 RS**  **p-values** | **LR v. L5 RS**  **p-values** | **L3 RS v. L5 RS**  **p-values** |
| --- | --- | --- | --- |
| Postnatal age at time of recording (days) | 0.5583 | 0.2345 | 0.5892 |
| Input resistance (MΩ) | 1.31e-26  *** | 3.07e-18  *** | 0.2798 |
| Input capacitance (pF) | 1.43e-30  *** | 6.11e-19  *** | 0.1321 |
| Membrane time constant (ms) | 0.3732 | 0.3506 | 0.7533 |
| Action potential threshold (mV) | 0.1873 | 0.1521 | 0.0250  * |
| Action potential amplitude (mV) | 4.82e-20  *** | 9.18e-07  *** | 0.1742 |
| Action potential width (ms) | 3.71e-06  *** | 1.25e-06  *** | 0.0914 |
| Spike frequency adaptation ratio | 2.94e-24  *** | 6.24e-15  *** | 0.5387 |
| Latency to first spike (ms) | 4.19e-15  *** | 1.37e-09  *** | 0.1748 |
| Rheobase (pA) | 1.24e-17  *** | 9.34e-12  *** | 0.0312  * |

**Supplementary Table 2. P-values for intrinsic property comparisons across cell type.**

All p-values for statistical significance between cell types for the intrinsic properties calculated and reported in Supplementary File 1 – Supplementary Table 1. Wilcoxon rank sum test was used for all comparisons.

**Supplementary Table 3. HD Cell Parameter Distributions**

| **Beta Distribution Parameters** | $\boldsymbol{a}$ | $\boldsymbol{b}$ | **Location** | **Scale** | **Mean** | **St. Dev.** |
| --- | --- | --- | --- | --- | --- | --- |
| $f_{max}$ | 2 | 3 | 0 | 175 | 70 | 40 |
| $f_{bg}$ | .6 | 150 | 0 | 150 | 0.6 | 0.8 |
| ATI | 2 | 3 | -10 | 150 | 50 | 30 |
| $\epsilon$ | 2 | 2 | 3 | 8 | 7 | 1.6 |

**Supplementary Table 3. Head direction cell parameter distributions.**

Parameter distributions for head direction cells that can be utilized with the scipy.stats.beta python function.
