# Supplementary material for: Thalamus and claustrum control parallel layer 1 circuits in retrosplenial cortex
Source: eLife. 2021 Jun 25;10:e62207. doi: 10.7554/eLife.62207 (PMC8233040; doi:10.7554/eLife.62207)
Supplement: Transparent reporting form [file elife-62207-transrepform.pdf]

eLIFE
1st Floor
24 Hills Road
Cambridge CB2 1JP, UK
P 01223 855340
W elifesciences.org
T @elife

eLife's transparent reporting form

We encourage authors to provide detailed information *within their submission* to facilitate the interpretation and replication of experiments. Authors can upload supporting documentation to indicate the use of appropriate reporting guidelines for health-related research (see EQUATOR Network), life science research (see the BioSharing Information Resource), or the ARRIVE guidelines for reporting work involving animal research. Where applicable, authors should refer to any relevant reporting standards documents in this form.

If you have any questions, please consult our Journal Policies and/or contact us: editorial@elifesciences.org.

Sample-size estimation

- You should state whether an appropriate sample size was computed when the study was being designed
- You should state the statistical method of sample size computation and any required assumptions
- If no explicit power analysis was used, you should describe how you decided what sample (replicate) size (number) to use

Please outline where this information can be found within the submission (e.g., sections or figure legends), or explain why this information doesn't apply to your submission:

Sample sizes reported in this paper are comparable to those used in recent slice electrophysiology and channelrhodopsion-assisted circuit mapping papers, and no statistical tests or analyses were used to predetermine sample size.

Replicates

- You should report how often each experiment was performed
- You should include a definition of biological versus technical replication
- The data obtained should be provided and sufficient information should be provided to indicate the number of independent biological and/or technical replicates
- If you encountered any outliers, you should describe how these were handled
- Criteria for exclusion/inclusion of data should be clearly stated
- High-throughput sequence data should be uploaded before submission, with a private link for reviewers provided (these are available from both GEO and ArrayExpress)

Please outline where this information can be found within the submission (e.g., sections or figure legends), or explain why this information doesn't apply to your submission:

All mouse lines and number of mice used in this study are detailed in the methods section. Numbers of cells used in each experiment in this study are detailed in both the results sections describing those findings and the figure legends for each experiment. Our only criteria for exclusion of data is stated in the methods section as: "cells with resting potentials more depolarized than -50 mV were not included in this study." Analysis-specific inclusion criteria are clearly stated within the methods where applicable.

eLife Sciences Publications, Ltd is a limited liability non-profit non-stock corporation incorporated in the State of Delaware, USA, with company number 5030732, and is registered in the UK with company number FC030576 and branch number BR015634 at the address 1st Floor, 24 Hills Road, Cambridge CB2 1JP | August 2014

Statistical reporting

- Statistical analysis methods should be described and justified
- Raw data should be presented in figures whenever informative to do so (typically when N per group is less than 10)
- For each experiment, you should identify the statistical tests used, exact values of N, definitions of center, methods of multiple test correction, and dispersion and precision measures (e.g., mean, median, SD, SEM, confidence intervals; and, for the major substantive results, a measure of effect size (e.g., Pearson's r, Cohen's d)
- Report exact p-values wherever possible alongside the summary statistics and 95% confidence intervals. These should be reported for all key questions and not only when the p-value is less than 0.05.

Please outline where this information can be found within the submission (e.g., sections or figure legends), or explain why this information doesn't apply to your submission:

Detailed statistical analysis methods are included in multiple places within the manuscript. Statistical analyses are separated within the methods section by experiment under the "Quantification and Statistical Analyses" sections. Additionally, the statistical test used for each analysis is stated within the results sections where each experiment and/or analysis is discussed as well as in all figure legends for each panel which includes statistical analysis. Values of N are also detailed in these results statements and figure legends. All quantifications of experimental data are presented as mean ± SEM, and error bars on most plots are SEM, unless otherwise stated (standard deviation is used in some of the modeling results to indicate degree of variance). This is also stated in the applicable legends. All p-values are reported with the exact value within the relevant results and figure legend text.

(For large datasets, or papers with a very large number of statistical tests, you may upload a single table file with tests, Ns, etc., with reference to sections in the manuscript.)

Group allocation

- Indicate how samples were allocated into experimental groups (in the case of clinical studies, please specify allocation to treatment method); if randomization was used, please also state if restricted randomization was applied
- Indicate if masking was used during group allocation, data collection and/or data analysis

Please outline where this information can be found within the submission (e.g., sections or figure legends), or explain why this information doesn't apply to your submission:

Experimental groupings are defined within the results text and legends of each figure, with Ns explicitly reported. Blinding or masking could not be used during data collection, as the locations of injection site and cell-type identities had to be verified by the experimenter each day to ensure each surgery and recording used in this study was representative of the pathways and cell types examined here. To avoid bias, recordings were alternated between cell types, and a standard and clearly defined set of tests and analyses was used to evaluate cell health and quantify results. These methods are clearly stated in the manuscript.

eLife Sciences Publications, Ltd is a limited liability non-profit non-stock corporation incorporated in the State of Delaware, USA, with company number 5030732, and is registered in the UK with company number FC030576 and branch number BR015634 at the address 1st Floor, 24 Hills Road, Cambridge CB2 1JP | August 2014

eLIFE
1st Floor
24 Hills Road
Cambridge CB2 1JP, UK
P 01223 855340
W elifesciences.org
T @elife

Additional data files ("source data")

- We encourage you to upload relevant additional data files, such as numerical data that are represented as a graph in a figure, or as a summary table
- Where provided, these should be in the most useful format, and they can be uploaded as "Source data" files linked to a main figure or table
- Include model definition files including the full list of parameters used
- Include code used for data analysis (e.g., R, MatLab)
- Avoid stating that data files are "available upon request"

Please indicate the figures or tables for which source data files have been provided:

We have included a Data and Software Availability section in the methods that provides information on model availability, neuronal reconstruction availability, and source data for experimental figures. It states "The LR neuron model utilized here is available on ModelDB (https://modeldb.yale.edu/260192), and the implementation of the Tsodyks-Markram model utilized here is also available from ModelDB (https://senselab.med.yale.edu/ModelDB/showmodel.cshtml?model=3815). Reconstructions will be available at http://neuromorpho.org/KeywordSearch.jsp and can be found by searching for the "Ahmed" archive. Source experimental data for figures 1-5, 7, Figure 1-Figure Supplement 1 & Supplementary File 1-Table 1 are provided."
